# Cleanliness

**Published:** 1897-11-06

**Authors:** 


### CLEANLINESS.

It is a matter worthy of observation that diseases are at the present moment current in all quarters of the globe, the preventive treatment of which, when reduced to its simplest terms, resolves itself into a mere enforcement of cleanliness, Yellow fever, plague, typhoid fever, and many others, are all recognised as infectious diseases, and attempts are being made on every hand to check their ravages by preventing the distribution of the infection; but everywhere it is being seen that infection defies such quarantine as is possible amid the conditions of modern life, that the most careful inquiry fails to discover all the cases, and that although isolation may be effective in preventing an outbreak when it is applied at the onset, it cannot do more than merely modify its course when the disease has once taken root. In the midst of all these epidemics it is obvious that certain groups of people escape while others suffer; and everywhere the same lesson is being taught that the essential step in fighting these diseases is the enforcement of cleanliness; cleanliness sometimes of person, sometimes of house, sometimes of food and drink, sometimes of municipality, as shown in scavenging, in drainage, in sewage disposal, and in water supply, but always cleanliness. This is, no doubt, old and commonplace; but old truths are all the more readily accepted when it is found that they are preached in many quarters and are applicable to many conditions. At Maidstone we find the many sanitary advantages which the town possesses nullified, and made of no avail by lack of cleanliness in the arrangements connected with the water supply. In Bombay and the surrounding districts we see plague cropping up in place after place, and always repeating the same history, attacking those who live among dirt, and sparing those whose surroundings are cleanly; in the southern part of the United States we see yellow fever breaking through all the quarantine by which the people have vainly attempted to control its progress, yet so held in check by cleanliness that its outbreaks are chiefly confined to towns where dirt prevails.

We do not want to preach a panacea, but when we find that a whole group of diseases can be practically held in check by one and the same set of sanitary measures, it does seem worth while to draw attention afresh to the importance of not allowing our sanitary endeavours to be diverted entirely to the treatment and isolation of individual cases, but rather of turning them into such channels as shall take away the terrors of many of these diseases, and shall lessen the dangers of the rest. We do not hesitate to say that there are in modern London an enormous number of dwellings whose condition is an absolute disgrace, and totally incompatible with healthy living. Much, no doubt, remains to be done in what we may call drainpipe sanitation; but some progress is being made, and, under the influence of public opinion, this is sure to go on. But hardly even a commencement has yet been made in raising the minimum standard of comfort at which it shall be permissible for human beings to live in this great city. People are allowed to pig together in conditions which are not only a danger to themselves, but a peril to the community, and the sanitary authorities are apparently helpless to restrain them. We have arrived at a point at which, if any further substantial improvement is to be effected in our sanitary condition, some one must boldly propose some very definite interference with home conditions, with personal liberty, with that grand possession of every Englishman, the right to have as many children as he likes, and to be as dirty as he pleases.

This is a matter which every politician shirks, and every party puts on one side, waiting for a more convenient occasion, waiting perhaps in hope that his enemy may take it up, for it is sure to be unpopular. But it must be taken up by someone if the health of the people is to be improved. At the root of the whole matter lies the question of over-crowding. As workhouses improve and sick wards are brought up to the level of the hospitals, as the hospitals are made more accessible by various parochial and church agencies, and now as the authorities show themselves willing to remove cases of infectious disease, the old people and the sick are to a large extent removed out of these tenements. This, however, does not leave more space, for it only enables the people to count on the removal and to crowd still more. Meanwhile, these hovels swarm with vermin, and dirt prevails to an indescribable degree, so that the district nurse goes about with elastics round her wrists that she may the more easily brush off the bugs that crawl upon her from every bed. This is the condition of affairs that exists in many a part of the greatest city of this great empire, and it is a disgrace to our times and to our civilisation.

